# Proactive antimicrobial stewardship with real-time microbiological alerts improves management of bloodstream infections

**DOI:** 10.1093/jacamr/dlaf182

**Published:** 2025-10-11

**Authors:** Arianna Di Marcello, Antonella Santoro, Vera Todisco, Erica Franceschini, Gabriella Orlando, Stefania Casolari, Adriana Cervo, Marianna Menozzi, Andrea Bedini, Davide Chemello, Mario Sarti, Jacopo Vecchiet, Katia Falasca, Cristina Mussini, Marianna Meschiari

**Affiliations:** Clinic of Infectious Diseases, Department of Medicine and Science of Aging, G. D'Annunzio University, Chieti, Italy; Clinic of Infectious Diseases, University of Modena and Reggio Emilia, Modena, Italy; Clinic of Infectious Diseases, University of Modena and Reggio Emilia, Modena, Italy; Clinic of Infectious Diseases, University of Modena and Reggio Emilia, Modena, Italy; Clinic of Infectious Diseases, University of Modena and Reggio Emilia, Modena, Italy; Clinic of Infectious Diseases, University of Modena and Reggio Emilia, Modena, Italy; Clinic of Infectious Diseases, University of Modena and Reggio Emilia, Modena, Italy; Clinic of Infectious Diseases, University of Modena and Reggio Emilia, Modena, Italy; Clinic of Infectious Diseases, University of Modena and Reggio Emilia, Modena, Italy; Clinic of Infectious Diseases, University of Modena and Reggio Emilia, Modena, Italy; Clinical Microbiology Laboratory, University of Modena and Reggio Emilia, Modena, Italy; Clinic of Infectious Diseases, Department of Medicine and Science of Aging, G. D'Annunzio University, Chieti, Italy; Clinic of Infectious Diseases, Department of Medicine and Science of Aging, G. D'Annunzio University, Chieti, Italy; Clinic of Infectious Diseases, University of Modena and Reggio Emilia, Modena, Italy; Clinic of Infectious Diseases, University of Modena and Reggio Emilia, Modena, Italy

## Abstract

**Introduction:**

This study aims to assess the impact of proactive Infectious Disease Specialist (IDS) interventions, in addition to standard antimicrobial stewardship (AMS) practices, triggered by real-time microbiological alerts, on improving the appropriateness and timeliness of antimicrobial prescriptions in hospitalized patients with bloodstream infections (BSIs).

**Methods:**

We conducted a prospective, single-center, pre-post interventional study at the University Hospital of Modena, Italy. Adult inpatients with monomicrobial BSIs between June 2022 and March 2023 were included. During the intervention phase (November 2022–March 2023), real-time microbiological alerts were automatically delivered to IDS consultants, who proactively reviewed therapy. Primary outcomes included the time to effective therapy (TTE) and the time to appropriate therapy (TTA). Secondary outcomes encompassed the duration of antimicrobial therapy, 14 and 30-day mortality from BSI, and hospital length of stay.

**Results:**

A total of 446 BSI episodes were analyzed (211 pre-intervention, 235 post-intervention). Post-intervention, the rate of appropriate therapy significantly increased (97.4% versus 76.2%, *P* < 0.001), and TTE was significantly shorter (0.63 versus 0.87 days, *P* = 0.022). No statistically significant reduction in TTA was observed (1.97 versus 2.37 days, *P* = 0.081). Early IDS intervention (<48 h) was associated with the shortest TTE and TTA. No significant differences were observed in mortality or hospital stay. Kaplan–Meier analysis showed a higher probability of receiving effective and appropriate therapy earlier in the post-intervention phase (log-rank test *P* = 0.014; 0.072, respectively). Subgroup analysis showed TTE improvements across MDR pathogens.

**Conclusions:**

A proactive intervention of IDS, based on automatic microbiological alert, in addition to routine AMS activities, is significantly associated with improved prescription appropriateness, reducing TTE.

## Introduction

Timely administration of appropriate antibiotic therapy plays a vital role in improving survival outcomes in patients with bloodstream infections (BSIs).^1^ Inappropriate empirical treatment has consistently been identified as an independent predictor of mortality in sepsis.^2,3^ In the current landscape of increasing antimicrobial resistance,^4^ national and international guidelines strongly advocate for the implementation of antimicrobial stewardship (AMS) programs.^5–7^ Rapid microbiological diagnostics, including fast microbiology techniques, represent essential tools that can be effectively integrated into AMS programs. When combined, these approaches have demonstrated a synergistic effect, enhancing the timeliness and appropriateness of antimicrobial therapy and ultimately improving key clinical outcomes in patients with BSIs.^8–31^ The involvement of the Infectious Disease Specialists (IDSs) in the treatment of BSIs has been associated with a significant increase in prescribing appropriateness and quality of patient care, while reducing consumption of antimicrobials and days of hospitalization.^32–37^

Although a few studies have explored the role of early AMS interventions in reducing time to effective (TTE) and appropriate therapy (TTA), the available evidence remains limited. In two Italian studies,^38,39^ the reassessment of clinical status and antimicrobial therapy at 72 h post-BSI onset was associated with improved appropriateness and reduced time to treatment initiation. However, these interventions were not implemented in real-time and lacked integration with automated microbiological alert systems. Similarly, two Korean studies investigated the use of computerized microbiological alert systems,^40,41^ showing improved likelihood of receiving effective and appropriate therapy and shortened TTE and TTA. Nonetheless, these systems operated largely in isolation from structured AMS teams and did not involve proactive, specialist-led clinical decision-making. While early AMS engagement and microbiological alerts show potential benefits, their independent implementation limits impact and generalizability; real-world evidence for an integrated, real-time model with active IDS involvement remains lacking.

This study addresses this gap by evaluating the impact of a proactive IDS intervention model, activated by real-time microbiological alerts, on the appropriateness and timeliness of antimicrobial prescriptions in patients with BSI.

## Materials and methods

### Study design

#### Setting and population

We conducted a prospective cohort, single-center, pre-post-intervention study at the University Hospital of Modena, a 1200-bed tertiary care facility in northern Italy. The study included all adult inpatients who developed a monomicrobial BSI between June 2022 and March 2023. Exclusion criteria were: age under 18 years; death, discharge, or transfer to another hospital within 48 h of BSI onset; blood cultures not meeting the criteria for BSI (negative or presence of contaminants); blood cultures collected in outpatient or day hospital settings; polymicrobial BSIs; and recurrent BSIs occurring within 30 days of a prior episode.

#### Study periods and interventions

The study was divided into two phases: a pre-intervention phase (June 2022–October 2022) and an intervention phase (November 2022–March 2023). The AMS team consisted of IDS, medical residents, a clinical microbiologist and a hospital pharmacist. During the pre-intervention phase, a standard AMS program combining restrictive and proactive strategies was implemented, with weekly audits and feedback. IDS consultations were reactive and non-systematic, occurring upon request or during routine AMS activities. During the intervention phase, real-time IDS interventions were triggered by daily microbiological alerts. These alerts were automatically generated once the microbiologist validated a positive blood culture and were promptly delivered to all IDS consultants via institutional email. Based on these alerts, IDS consultants proactively enrolled patients and contacted the relevant clinical teams to immediately review and optimize antibiotic therapy. Interventions included initiation of empirical or targeted therapy, re-evaluation of ongoing treatment after antibiogram availability (e.g. confirmation, de-escalation, escalation, or switch). Even in cases where in vitro susceptibility suggested the use of antibiotics that might be considered inappropriate, such as when the pathogen expressed clinically relevant AMPC, the IDS accounted for these resistance mechanisms to guide therapy selection. All IDS interventions were carried out according to international AMS guidelines,^8^ and were available Monday to Friday, from 8:30 a.m. to 4:30 p.m., excluding public holidays. We showed the microbiological workflow in Figure S1 (available as Supplementary data at *JAC-AMR* Online).

### Data collection

Baseline demographic, epidemiological, and clinical data were collected at the time of enrolment. The severity of clinical presentation at the onset of BSI was assessed using the Pitt bacteraemia score.^42^ Data were extracted from both electronic and paper medical records and entered into a dedicated database using tablet devices, covering the period from hospital admission through to 90 days post-discharge (including any readmissions within that timeframe). We selected all blood cultures that met the criteria for monomicrobial BSI. For each patient, monomicrobial BSIs caused by different bacterial species were considered as different episodes; recurrences with the same pathogen isolated within 30 days of the initial positive blood culture were classified as part of the same episode. Blood cultures collected within 24 h prior to antibiotic initiation were considered as taken before treatment. We collected 90-day mortality and rehospitalization data through a combination of hospital records, electronic health databases, and direct follow-up with patients or their caregivers. Rehospitalizations were identified through review of hospital admission records within the 90-day follow-up period. Patients lost to follow-up were censored at the last known contact date.

### Definitions and outcomes

Bloodstream infection (BSI): Isolation of the same bacterial species from at least two separate blood culture sets drawn from different sites, or isolation of a recognized pathogen from at least one blood culture set. Monomicrobial episode: Isolation of a single microbial species in blood cultures. Polymicrobial episode: Isolation of more than one microbial species from blood cultures within two days (day 0, 1, or 2) of the initial positive culture.

Effective therapy: Antimicrobial treatment for which the isolated pathogen shows *in vitro* susceptibility, referring to both the empirical and targeted regimen. Appropriate therapy: Appropriate antimicrobial therapy was defined by the IDS according to pathogen susceptibility in vitro, clinical presentation and current guidelines, ensuring the narrowest effective spectrum (taking into account patient allergies and co-infections) as well as optimal pharmacokinetic/pharmacodynamic properties (ensuring a sufficient antibiotic concentrations at the site of infection for an adequate duration to effectively kill or inhibit bacterial growth).

TTE (time to effective therapy): Time (in days) from blood culture collection to the start of effective antimicrobial therapy. TTA (time to appropriate therapy): Time from blood culture collection to the start of appropriate antimicrobial therapy.

De-escalation (eucast definition): A deliberate switch from broad-spectrum empirical therapy to a narrower-spectrum agent with equivalent or greater efficacy, based on microbiological results and clinical judgment. Escalation: Adjustment of antimicrobial treatment by broadening the spectrum, typically due to clinical deterioration or identification of resistant pathogens. Switch: A change in the antibiotic molecule (e.g. to a different class) or route of administration (e.g. from intravenous to oral), based on clinical and pharmacological considerations.

Sepsis (sepsis-3 definition 2016): Life-threatening organ dysfunction caused by dysregulated host response to infection (Key Clinical Criteria: SOFA score increase ≥2 points; infection confirmed or suspected). Septic shock: subset of sepsis with circulatory and metabolic abnormalities (Key Clinical Criteria: vasopressor needed to maintain Mean Arterial Pressure ≥65 mmHg and serum lactate >2 mmol/L after adequate fluid resuscitation).

Outcomes were compared between the two periods (pre-intervention and intervention). Primary outcomes were TTE therapy and time to appropriate therapy. Secondary outcomes encompassed: duration of antimicrobial therapy; type of AMS intervention (confirmation of correct therapy, de-escalation, escalation, and therapy switch); hospital length of stay; 14, 30 and 90-day all-cause mortality; rehospitalization at 90 days.

### Microbiological analysis

Blood samples were inoculated in aerobic and anaerobic Bactec and incubated at 35°C in the automated blood culture systems. When growth was detected, species level identification of the infecting pathogens was conducted in blood culture broths using MALDI-TOF MS testing.^43^ All specimens were also processed according to the standard procedure that includes antimicrobial susceptibility testing by rapid methods (RAST) and by conventional culture-based methods. Results were interpreted in accordance with the EUCAST clinical breakpoints.^44^

### Statistical analysis

Data are expressed as mean and standard deviation (SD) for continuous variables, and they were compared by Student’s *t*-test or by analysis of variance, as appropriate, according to pre-intervention and intervention or according to the different types of alerts (RAST, species identification or definitive antibiograms). Likewise, categorical variables are expressed as number and percentage and were analyzed using the χ^2^; the Fisher’s exact tests were applied when expected frequencies in some cells of the contingency table were very low. Moreover, time to implementation of effective and appropriate antimicrobial therapy was compared between the pre-intervention and the intervention phase using Kaplan–Meier survival analysis with a log-rank test. Post-hoc subgroup analyses comparing early and late intervention were conducted using the same analytical methods. A two-way ANOVA with post-hoc test was employed to investigate the main and interaction effects of intervention and antimicrobial resistance on TTE and TTA.

All *P*-values were two-sided and *P*-values <0.05 were considered statistically significant. SPSS Statistics version 28.0 was used for all statistical calculations.

## Results

A total of 446 BSI episodes occurring in 421 patients were included in the analysis: 211 (47.3%) occurred in 193 patients during the pre-intervention phase; 235 (52.7%) occurred in 228 patients during the intervention phase.

### Baseline patient and microbiological characteristics

Patients were predominantly male, with a mean age of 72 ± 15.6 years and a mean Charlson Comorbidity Index of 2 ± 2.0. Age, sex, and comorbidities were similar between groups. The severity of bacteraemia, assessed by the Pitt score, and sepsis rates were also comparable (Table 1).

**Table 1.** Baseline characteristics of the patients and BSIs included from each study period

Variables *n*	Total patients, *n* = 421 (100%)	Pre-Intervention, *n* = 193 (45.8%)	Intervention, *n* = 228 (54.2%)	*P* value
Age (in years ± SD)	72.3 (±15.6)	73.2 (±16.1)	71.5 (±15.1)	0.256
Female gender (%)	184 (43.7)	83 (43)	101 (44.3)	0.844
Service department (%)				
Medical	302 (71.7)	144 (74.6)	158 (69.3)	0.216
Surgical	59 (14.0)	29 (15)	30 (13.2)	0.216
Oncohematological	43 (10.2)	14 (7.3)	29 (12.7)	0.216
ICU	17 (4.1)	6 (3.1)	11 (4.8)	0.216
Site of acquisition (%)				
Community-acquired	379 (90.0)	175 (90.7)	204 (89.5)	0.166
Long-term Care Facilities	21 (5.0)	6 (3.1)	15 (6.6)	0.166
Hospital-onset	21 (5.0)	12 (6.2)	9 (3.9)	0.166
Comorbidities (%)				
Myocardial infarction	43 (10.2)	23 (11.9)	20 (8.8)	0.334
Congestive heart failure	34 (8.1)	23 (11.9)	11 (4.8)	0.011
Peripheral vascular disease	52 (12.4)	29 (15)	23 (10.1)	0.138
Diabetes mellitus				
Uncomplicated	75 (17.8)	31 (16.1)	44 (19.3)	0.594
Complicated (organ damage)	17 (4.0)	9 (4.7)	8 (3.5)	0.594
Stroke/Transient Ischaemic Attack	31 (7.3)	12 (6.2)	19 (8.3)	0.457
Dementia	61 (14.5)	26 (13.5)	35 (15.4)	0.677
Chronic Obstructive Pulmonary Disease	40 (9.5)	22 (11.4)	18 (7.9)	0.245
Rheumatological disease	17 (4.0)	13 (6.7)	4 (1.8)	0.023
Hemiplegia/paraplegia	2 (0.5)	0 (0)	2 (0.9)	0.502
Liver disease				
Mild	15 (3.6)	5 (2.6)	10 (4.4)	0.644
Moderate/severe	46 (10.9)	21 (10.9)	25 (11.0)	0.644
Chronic Renal Failure	87 (20.7)	35 (18.1)	52 (22.8)	<0.001
Solid tumour				
Localized	48 (11.4)	22 (11.5)	26 (11.4)	0.963
Metastatic	28 (6.7)	12 (6.3)	16 (7.0)	0.963
Leukaemia	18 (4.3)	10 (5.2)	8 (3.5)	0.472
Lymphoma	25 (5.9)	8 (4.1)	17 (7.5)	0.214
HIV/AIDS	6 (1.4)	2 (1.0)	4 (1.8)	0.692
Charlson Comorbidity Index (±SD)	2.54 (±2.1)	2.42 (±2.0)	2.65 (±2.1)	0.248
Solid organ transplantation	35 (8.3)	15 (7.8)	20 (8.8)	0.727
Neutropenia	17 (4.0)	7 (3.6)	10 (4.4)	0.806
Hematopoietic Stem Cell Transplantation	1 (0.2)	1 (0.5)	0 (0)	
Extrinsic risk factors: Devices (%)				
CVC/PICC	88 (21.0)	42 (21.8)	45 (19.7)	0.630
Endovascular devices (stent, grafts)	12 (2.9)	5 (2.6)	7 (3.1)	0.781
Joint prosthesis/orthopedic implants	21 (5.0)	8 (4.1)	13 (5.7)	0.508
Cardiac devices (PM, ICD, valve prostheses)	38 (9.02)	16 (8.3)	22 (9.6)	0.528
Vesical catheter	108 (14.1)	49 (25.4)	59 (25.9)	0.911
Miscellaneous^a^	90 (21.4)	52 (26.9)	38 (16.7)	0.012

Variables *n* (%)	Total BSI, *n* = 446 (100%)	Pre-Intervention, *n* = 211 (47.3%)	Intervention, *n* = 235 (52.7%)	*P* value
Severity of illness				
Pitt score (±SD)	0.96 (±1.3)	0.91 (±1.3)	1 (±1.3)	0.463
Sepsis/septic shock	217 (48.7)	96 (45.5)	121 (51.5)	0.218
Site of infection				
Primary bacteraemia	50 (11.2)	22 (10.4)	28 (11.9)	0.547
Catheter-related infection	54 (12.1)	31 (14.7)	23 (9.8)	0.139
Pneumonia	44 (9.9)	22 (10.4)	22 (9.3)	0.519
Intra-abdominal infection	114 (25.6)	52 (24.6)	62 (26.4)	0.744
Urinary tract infection	139 (31.2)	70 (33.2)	69 (29.4)	0.412
Others^b^	45 (10.1)	14 (6.6)	31 (13.2)	0.027
Pathogen				
Gram-positive bacteria	161 (36.1)	64 (30.3)	97 (41.3)	0.018
*Staphylococcus aureus*	53 (11.9)	20 (9.5)	33 (14.0)	
*Enterococcus species*	45 (10.1)	16 (7.6)	29 (12.3)	
*Coagulase-negative staphylococci*	29 (6.5)	19 (9.0)	10 (2.4)	
Gram-negative bacteria	274 (61.4)	140 (66.4)	134 (57.0)	0.05
*Enterobacterales*	231 (51.8)	129 (61.1)	102 (43.3)	
*Pseudomonas aeruginosa*	25 (5.6)	9 (4.3)	16 (6.8)	
Yeast	11 (2.5)	7 (3.3)	4 (1.7)	0.363
Resistance mechanism				
Absent	343 (76.9)	153 (72.5)	190 (80.9)	0.043
MRSA/MRSE	31 (7.0)	17 (8.1)	14 (6.0)	0.457
ESBL/AmpC-producing	57 (12.8)	34 (16.1)	23 (9.8)	0.048
Carbapenemase	9 (2.0)	3 (1.4)	6 (2.6)	0.509
VRE	4 (0.9)	2 (0.9)	2 (0.9)	1.00
Azole resistance	2 (0.4)	2 (0.9)	0	0.223

CVC, Central Venous Catheter; ESBL, Extended-spectrum Beta-Lactamase; ICD, Implantable Cardioverter-Defibrillator; PICC, Peripherally Inserted Central Catheter; PM, pacemaker; SD, Standard Deviation; VRE, Vancomycin-resistant Enterococcus.

^a^Surgical drains, endotracheal tubes, thoracostomy/tracheostomy tubes, peritoneal dialysis catheters, peritoneal/ventricular shunts.

^b^Skin and soft tissue infection, infective endocarditis, bone and joint infection, CNS infection, oropharyngeal infection.

Pathogen distribution differed slightly: Gram-positive pathogens were more frequent in intervention phase (41.3% versus 30.3%, *P* = 0.018), while Gram-negative pathogens predominated pre-intervention (66.4% versus 57.0%, *P* = 0.05). MDR organisms were isolated in 23.1% of cases, including 7.0% MRSA, 0.9% VRE, 12.8% ESBL-/AmpC-producing *Enterobacterales*, 2.0% carbapenemase-producing *Enterobacterales* (CPE), and 0.4% azole-resistant yeasts.

### AMS intervention and clinical outcomes

Among the 446 BSI included, different type of AMS interventions were implemented: confirmation of correct therapy in 33.2%, de-escalation in 40.4%, escalation in 15.9%, and therapy switch in 7.6% of cases. Confirmation of correct therapy was significantly more frequent in the intervention group compared with the pre-intervention group (37.4% versus 28.4%, *P* = 0.045). No significant differences were observed in the rates of de-escalation, escalation, or therapy switch between the two groups. Nearly all patients received effective therapy (99.6%), with no difference between groups (99.0% pre-intervention versus 100% intervention group, *P* = 0.223). However, the proportion of patients receiving appropriate therapy was significantly higher in intervention group (97.4% versus 76.2%, *P* < 0.001). TTE therapy was significantly shorter in the intervention group (0.63 ± 0.92 versus 0.87 ± 1.2 days, *P* = 0.022), while time to appropriate therapy showed a non-significant trend towards improvement (1.97 ± 2.0 versus 2.37 ± 2.4 days, *P* = 0.081). In most cases the transition from effective to appropriate therapy was made promptly following the AMS review, once definitive microbiological identification and susceptibility testing results became available. This typically occurred within 24–48 h of blood culture positivity.

No significant differences were observed between groups in terms of antibiotic duration (12.8 ± 10.9 versus 13.4 ± 17.0 days, *p* = 0.704), length of hospitalization (26.9 ± 27.7 versus 29.8 ± 30.2 days, *P* = 0.301), or clinical outcomes. Specifically, 14-day mortality was 8.1% overall (9.8% intervention group versus 6.2% pre-intervention, *P* = 0.169), 30-day mortality was 14.1% (17.1% versus 10.9%, *P* = 0.077), and 90-day mortality was 24.9% (26.9% versus 21.8%, *P* = 0.156). Rehospitalization at 90 days occurred in 29.4% of cases, with no significant difference between groups (27.2% versus 31.8%, *P* = 0.300) (Table 2).

**Table 2.** Primary and secondary study outcomes

	Total BSI, *n* = 446 (100%)	Pre-Intervention, *n* = 211 (47.3%)	Intervention, *n* = 235 (52.7%)	*P*-value
Type of Antimicrobial Stewardship Intervention				
Confirmation	148 (33.2)	60 (28.4)	88 (37.4)	0.045
De-escalation	180 (40.4)	89 (42.2)	91 (38.7)	0.499
Escalation	71 (15.9)	39 (18.5)	32 (13.6)	0.195
Therapy switch	34 (7.6)	12 (5.7)	22 (9.4)	0.157
AMS outcomes				
Effective therapy in total (%)	444 (99.6)	209 (99)	235 (100)	0.223
Appropriate therapy in total (%)	389 (87.4)	160 (76.2)	229 (97.4)	<0.001
TTE therapy (in days ± SD)	0.74 (±1.1)	0.87 (±1.2)	0.63 (±0.92)	0.022
Time to appropriate therapy (in days ± SD)	2.13 (±2.2)	2.37 (±2.4)	1.97 (±2.0)	0.081
Duration of antibiotic therapy (in days ± SD)	13.1 (±13.9)	13.4 (±17.0)	12.8 (±10.9)	0.704
Clinical outcomes				
Mortality at 14 days	36 (8.1)	13 (6.2)	23 (9.8)	0.169
Mortality at 30 days	63 (14.1)	23 (10.9)	40 (17.1)	0.077
Mortality at 90 days	111 (24.9)	46 (21.8)	65 (26.9)	0.156
Rehospitalization at 90 days (%)	131 (29.4)	67 (31.8)	64 (27.2)	0.300
Length of hospitalization (in days ± SD)	28.3 (±28.9)	29.8 (±30.2)	26.9 (±27.7)	0.301

SD, Standard Deviation.

**Figure 1.**
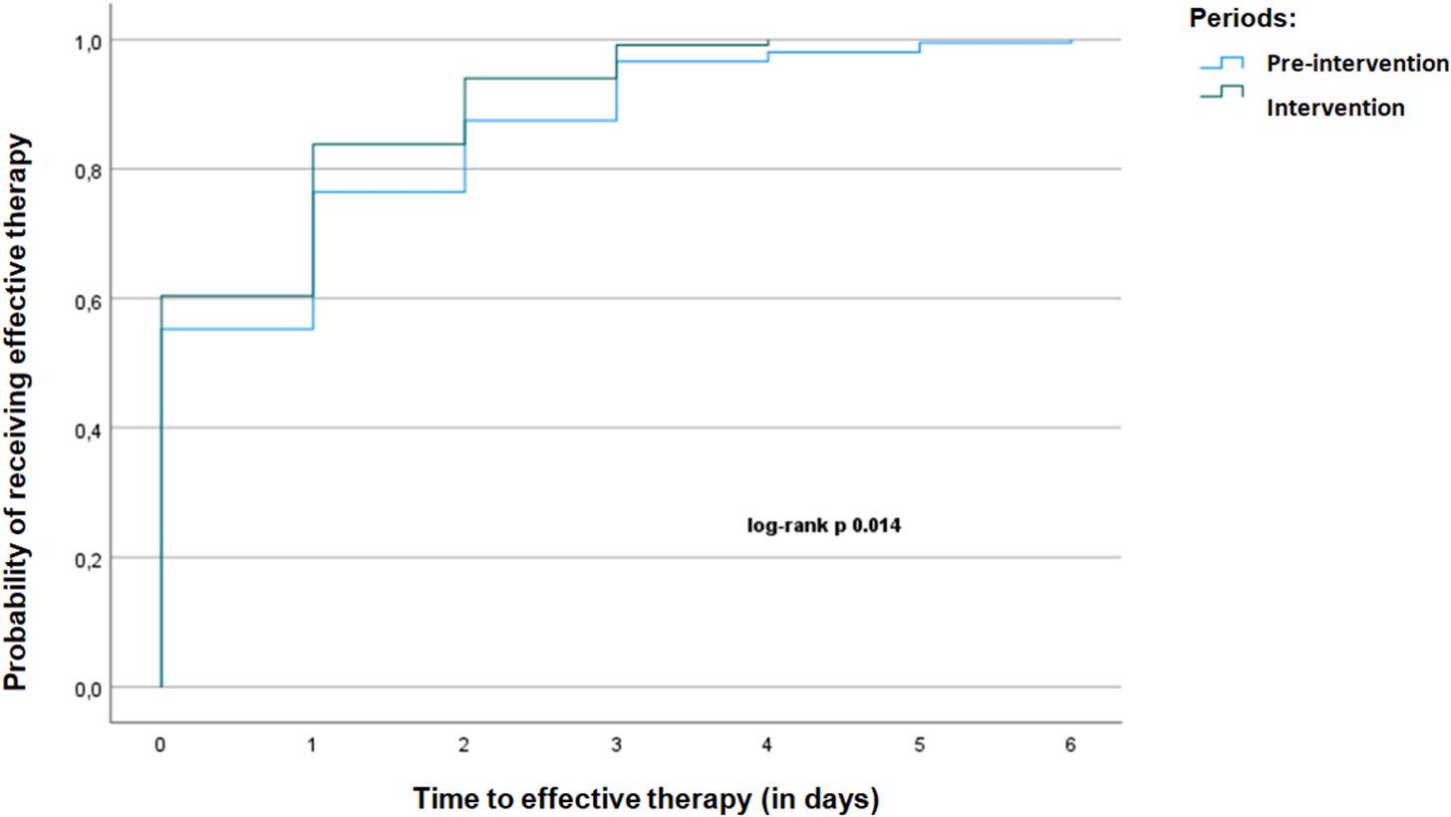
Kaplan–Meier curve comparing TTE between the pre-intervention and intervention periods.

**Figure 2.**
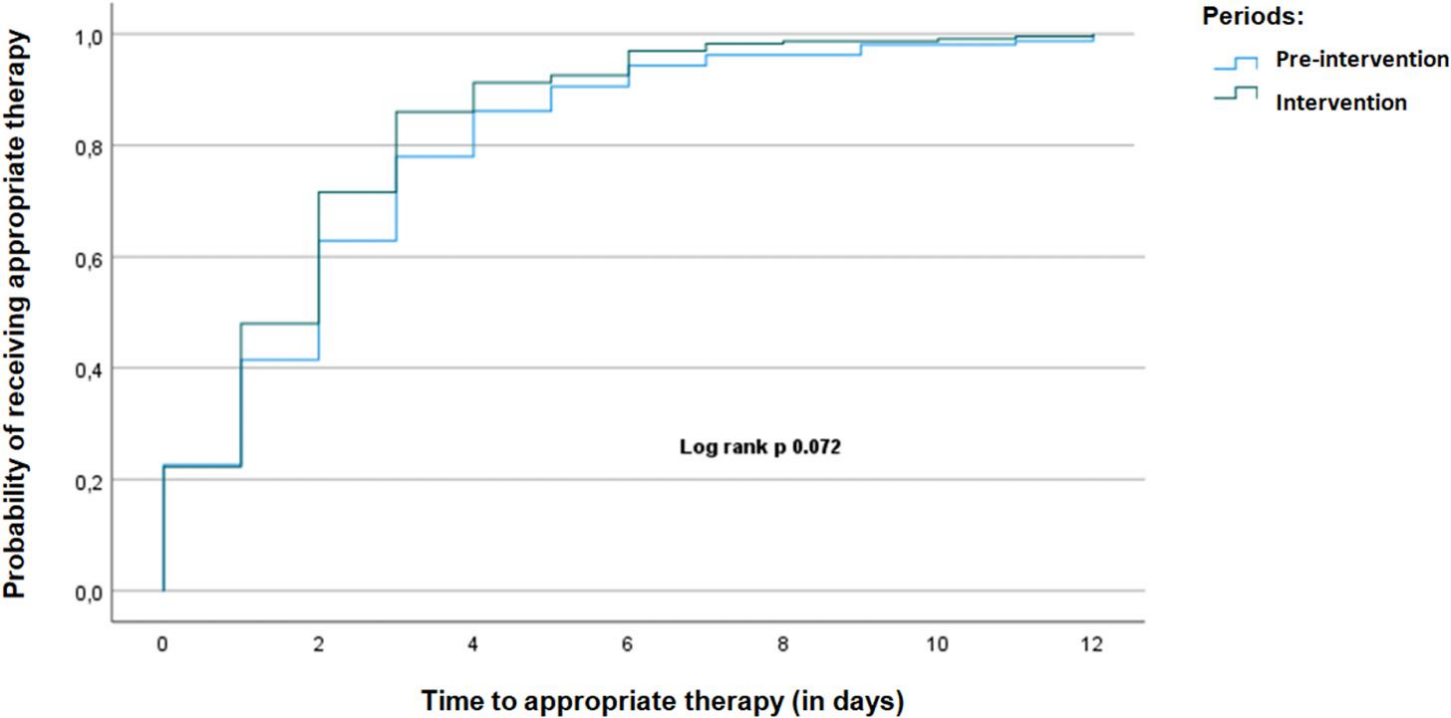
Kaplan–Meier curve comparing TTA between the pre-intervention and intervention periods.

The Kaplan–Meier analysis demonstrated a significantly shorter TTE therapy in the intervention group compared with the pre-intervention group (log-rank *P* = 0.014). The probability of receiving effective therapy increased more rapidly in the intervention period, with most patients achieving effective therapy within the first 2 days of bacteraemia onset (Figure 1). Regarding appropriate therapy, there was a clear trend toward statistical significance in both TTA and the Kaplan–Meier analysis, favouring the intervention group (log-rank *P* = 0.072) (Figure 2).

### Subgroup analyses

#### Early versus late intervention in the intervention group

We further analysed outcomes within the intervention group based on the timing of IDS intervention (Table S1). Early intervention, defined as occurring within 48 h of blood culture collection, was associated with significantly shorter TTE therapy (TTE: 0.37 ± 0.5 versus 0.87 ± 1.1 days, *P* < 0.001) and time to appropriate therapy (TTA: 1.31 ± 1.5 versus 2.59 ± 2.1 days, *P* < 0.001) compared with late intervention (≥48 h). Length of hospitalization and mortality rates at 14, 30, and 90 days were similar between groups.

#### Impact of antimicrobial resistance on time to therapy

A sub analysis by pathogen resistance profiles revealed significant differences in TTE according to antimicrobial resistance patterns in both the pre-intervention and intervention groups (Table 3, *P*-values are calculated in reference to the non-resistant strains). In the pre-intervention phase, patients with CPE had the longest TTE (2.0 ± 2.6 days), compared with those with no resistance (0.7 ± 1.1 days, *P* = 0.001). A similar trend was observed in intervention group, with CPE cases again showing the longest TTE (1.8 ± 1.5 days, *P* = 0.001). TTA was also influenced by resistance patterns, showing a borderline significant association in the pre-intervention group (*P* = 0.096), though this was not observed in intervention phase (*P* = 0.449). Overall, antimicrobial resistance significantly affected both TTE and TTA (*P* < 0.001 and *P* = 0.052, respectively).

**Table 3.** Sub analysis of TTE and TTA per MDR pathogens in the pre-intervention and intervention groups

	No resistance	MRSA/MRSE	ESBL/AmpC	Carabapenemase	VRE	*P*-value
Pre-intervention group						
TTE (in days ± SD)	0.7 (±1.1)	1.6 (±1.2)	1.2 (±1.3)	2.0 (±2.6)	1.0 (±1.4)	**0**.**001**
TTA (in days ± SD)	2.5 (±2.4)	1.9 (±1.2)	1.8 (±1.9)	6.0 (±5.5)	2.0 (±2.8)	0.096
Intervention group						
TTE (in days + SD)	0.54 (±0.9)	0.57 (±0.9)	1.0 (±0.6)	1.8 (±1.5)	1.5 (±2.1)	**0**.**001**
TTA (in days + SD)	2.1 (±2.1)	1.4 (±1.4)	1.4 (±1.2)	1.8 (±1.5)	2.0 (±1.4)	0.449

AMPc, AmpC Beta-lactamases; ESBL, Extended spectrum Beta-Lactamase; MRSA, Methicillin-resistant *Staphylococcus aureus*; MRSE, Methicillin-resistant Staphylococcus epidermidis; SD, Standard Deviation; TTA, time to appropriate therapy; TTE, time to effective therapy; VRE, Vancomycin-resistant Enterococcus.

## Discussion

Our study showed that proactive, real-time AMS interventions based on computerized microbiology alerts improved appropriate antibiotic prescribing, reduced TTE and increased the overall prevalence of active therapy in hospitalized patients with BSIs, beyond standard AMS practices. The lack of a statistically significant reduction in TTA may reflect the effect of the pre-existing stewardship program in the pre-intervention phase. Baseline TTA was likely already reduced, limiting the potential for further improvement during the intervention phase—a ‘ceiling effect’ that may explain why TTA did not change significantly, even though the intervention accelerated the achievement of effective therapy.

Our model provides proof of concept that integrating antibiotic and diagnostic stewardship enhances treatment optimization, resistance monitoring, and responsible antibiotic use, ultimately improving patient care.^45–51^ A recent open-label, cluster-randomized trial in three Swiss hospitals showed no significant improvement in appropriate antibiotic selection regarding agent choice and duration.^52^ The key advantage of our integrated system is its feasibility, utilizing standard software commonly available in clinical microbiology labs without needing addiction tools or external IT platforms.

Our strategy is especially relevant in high-MDR settings, where timely appropriate therapy is challenging; patients with MDR organisms were more likely to receive inappropriate empiric therapy, increasing mortality.^53,54^

The higher number of patients receiving effective initial therapy during the intervention phase suggests our integrated model indirectly improves medical education. While IDS play a crucial role in BSI management,^55–58^ their impact is greatest within a proactive, multidisciplinary AMS team.

Appropriate therapy switches typically occurred within 24–48 h after blood culture positivity, guided by AMS and definitive microbiological results. Early IDS review improved TTE and TTA, while later interventions were linked to shorter antibiotic courses. Late IDS intervention may offer practical advantages. The availability of antibiogram results allows for safer and more targeted therapy, avoiding unnecessarily broad or prolonged empirical regimens. Additionally, evaluating the patient’s clinical response after several days of treatment enables more accurate adjustment of the therapeutic course. Finally, integrating microbiological data with clinical evolution supports informed decisions regarding therapy duration in accordance with guidelines, potentially reducing the risk of prolonged antibiotic use.

Systematic antimicrobial prescription review occurred only during the intervention phase, indicating that differences between early and late intervention likely reflect timely IDS involvement rather than the alert system alone. While correct therapy confirmation improved post-intervention, factors like concurrent education, guideline updates, infection severity, and pathogen distribution may have influenced the likelihood of receiving correct therapy. Moreover, the observed increase in Gram-positive pathogens during the intervention period may have contributed to a lower rate of empiric therapy coverage, particularly for MRSA. This underscores the need to tailor empiric regimens to local microbiological trends; AMS should account for pathogen distribution shifts to ensure timely and effective coverage of both Gram-positive and Gram-negative infections.

Few studies have assessed the clinical impact of early AMS interventions on BSI,^38–41^ with a key limitation being that IDS did not receive direct computerized microbiological alerts. In Murri *et al.*,^38,39^ IDS were contacted by the microbiologist by phone after a pathogen was isolated from the blood culture and without preliminary susceptibility data; moreover patients admitted to haematology, ICU and in the Emergency Department were excluded. In Kim *et al.*, alerts went to wards, with IDS supervising non-specialist-initiated therapies.^40,41^ Our study is the first in which the computerized microbiological alert is sent directly and in real-time to the IDS, promoting a proactive AMS approach. Consistent with previous studies, no significant differences were observed in survival or other secondary clinical outcomes.^38^ Thirty-day mortality was higher in the intervention phase, likely because over 99% of patients in both phases received effective empirical broad-spectrum therapy from the start. This may have limited the impact of AMS on hard outcomes (mortality, hospital stay, or therapy duration). Under these conditions, proactive AMS may primarily influence antimicrobial optimization practices rather than exert a direct impact on survival or hospitalization metrics. Mortality differences could also reflect case-mix confounding or chance rather than a true intervention effect. In such settings, AMS programs mainly optimize antimicrobial use; reduce resistance and toxicity, rather than directly affecting short-term outcomes like mortality. Since TTA impacts hospital ecology and resistance control, these secondary outcomes should be included in future research. Our model also promotes ongoing medical staff education via integrated diagnostic stewardship and real-time prescribing feedback.^59^ This approach reinforces the critical role of the AMS team and promotes the adoption of rational antimicrobial strategies. Additional interventions, together with this proactive IDS intervention model, may significantly reduce the time to appropriate antibiotic therapy: such as screening of rectal colonization, evaluation of risk factors,^60^ and the use of rapid microbiological diagnostics.^61^

Our single-center, small-sample pre-post study has limited generalizability and potential selection bias. Kaplan–Meier analyses were unadjusted for confounders (e.g. source of infection, severity scores), with future adjustments planned. Although secular trends other concurrent improvements in AMS practice cannot be fully excluded, the short study period and the absence of major programmatic or laboratory changes reduce this risk. Rapid resistance gene testing was selectively applied, particularly in septic patients admitted to high-risk wards and in cases involving CPE. The lack of rapid molecular resistance gene testing may represent a relevant limitation, as it could further improve time to appropriate therapy in MDR infections. In low-prevalence settings, rapid phenotypic methods like RAST may be a cost-effective alternative, warranting further comparative studies.

Moreover, the AMS service was not available around the clock. Finally longer-term impact of this proactive IDS intervention on additional outcomes (emergence of antibiotic resistance or *Clostridium difficile* infection, overall antibiotic consumption and healthcare costs) was not evaluated.

### Conclusions

Prompt, proactive IDS intervention alongside routine AMS facilitates earlier initiation of effective treatments. Continuous feedback and 48-h therapy re-evaluation by IDS influence antimicrobial therapy duration. Our findings indicate that integrating AMS with diagnostic stewardship improves prescription appropriateness. A key strength of our study was the direct, real-time delivery of computerized microbiological alerts to IDS.

For future studies, it would be useful the Desirability of Outcome Ranking for the Management of Antimicrobial Therapy (DOOR MAT), a patient-centric system that allows multiple outcomes to be considered.^62,63^

The clinical benefits of reducing TTE remain unclear and require further study. Future research should identify conditions where TTE improvements impact outcomes like mortality, length of stay, or treatment duration. Pragmatic RCTs linking AMS-driven antibiotic appropriateness to resistance prevalence are a priority.^64^

## Supplementary Material

dlaf182_Supplementary_Data

## Supplementary data

Figure S1 and Table S1 are available as Supplementary data at *JAC-AMR* Online.
