# Supplementary material for: Proactive antimicrobial stewardship with real-time microbiological alerts improves management of bloodstream infections
Source: JAC Antimicrob Resist. 2025 Oct 11;7(5):dlaf182. doi: 10.1093/jacamr/dlaf182 (PMC12514464; doi:10.1093/jacamr/dlaf182)
Supplement: dlaf182_Supplementary_Data [file dlaf182_supplementary_data.doc]

Supplementary Materials

***Supplementary Figure 1: Microbiological workflow***

*ID: identification; RAST: Rapid Antimicrobial Susceptibility Testing; IDS: Infectious Diseases Specialist*

***Supplementary Table 1: Outcomes in intervention group basing on time of intervention.***

| **Variables** | **Total BSI** | **Early intervention**  **(< 48 hours)** | **Late intervention**  **(≥48 hours)** | **p-value** |
| --- | --- | --- | --- | --- |
| **Type of intervention:**  - Correct therapy  - De-escalation  - Escalation  - Therapy switch | 88 (37.4)  91 (38.7)  32 (13.6)  22 (9.4) | 46 (19.6)  37 (15.7)  21 (8.9)  10 (4.3) | 42 (17.9)  54 (23.0)  11 (4.7)  12 (5.1) | 0.06 |
| **TTE** (in days ± SD) | 0.8 (± 3.3) | 0.37 (± 0.5) | 0.87 (±1.1) | <0.001 |
| **TTA** (in days ± SD) | 2.17 (± 3.7) | 1.31 (±1.5) | 2.59 (±2.1) | <0.001 |
| **Length of hospitalisation** (in days ± SD) | 24.9 (± 22.5) | 28.5 ( ± 33.1) | 25.5 (±21.1) | 0.419 |
| **Duration of antibiotic therapy** (in days ± SD) | 12.6 (± 10.5) | 14.3 (± 13.7 ) | 11.73 ( ± 6.4 ) | 0.04 |
| **Mortality at 14 days** | 23 (9.8) | 13 (5.5) | 10 (4.3) | 0.516 |
| **Mortality at 30 days** | 40 (17.1) | 19 (8.1) | 21 (9.0) | 0.863 |
| **Mortality at 90 days** | 65 (26.9) | 31 (13.2) | 34 (14.5) | 0.772 |
| **Rehospitalization at 90 days** | 64 (27.2) | 32 (13.7) | 32 (13.7) | 1.00 |

*SD: Standard Deviation; TTE: time to effective therapy; TTA: time to active therapy.*
